# Overexpression of *SAMDC1* gene in *Arabidopsis thaliana* increases expression of defense-related genes as well as resistance to *Pseudomonas syringae* and *Hyaloperonospora arabidopsidis*

**DOI:** 10.3389/fpls.2014.00115

**Published:** 2014-03-27

**Authors:** Francisco Marco, Enrique Busó, Pedro Carrasco

**Affiliations:** ^1^Departament de Biologia Vegetal, Facultat de Farmàcia, Universitat de ValènciaValència, Spain; ^2^Departament de Bioquímica i Biologia Molecular, Facultat de Ciències Biològiques, Universitat de ValènciaValència, Spain

**Keywords:** biotic stress, spermine, jasmonate, polyamines, stress response and stress tolerance

## Abstract

It has been previously described that elevation of endogenous spermine levels in Arabidopsis could be achieved by transgenic overexpression of S-Adenosylmethionine decarboxylase (*SAMDC*) or Spermine synthase (*SPMS*). In both cases, spermine accumulation had an impact on the plant transcriptome, with up-regulation of a set of genes enriched in functional categories involved in defense-related processes against both biotic and abiotic stresses. In this work, the response of *SAMDC1*-overexpressing plants against bacterial and oomycete pathogens has been tested. The expression of several pathogen defense-related genes was induced in these plants as well as in wild type plants exposed to an exogenous supply of spermine. *SAMDC1*-overexpressing plants showed an increased tolerance to infection by *Pseudomonas syringae* and by *Hyaloperonospora arabidopsidis*. Both results add more evidence to the hypothesis that spermine plays a key role in plant resistance to biotic stress.

## Introduction

Polyamines (PAs) constitute a group of low molecular weight aliphatic amines, whose most widespread forms in living organisms are the diamine putrescine (Put), the triamine spermidine (Spd) and the tetraamine spermine (Spm). In plants, PAs have been implicated as key players in growth and development processes, as well as in the response to biotic and abiotic stresses (Kusano et al., 2008; Alcázar et al., 2010; Takahashi and Kakehi, 2010). Intracellular PA levels in plants are mostly regulated by anabolic and catabolic processes, as well as by their conjugation to hydroxycinnamic acids and macromolecules like proteins and DNA.

PA biosynthesis pathway has been well characterized in *A. thaliana* (Alcazar et al., 2006). In this species, PA synthesis is initiated with Put synthesis from aminoacid arginine by the sequential action of arginine decarboxylase (ADC; EC 4.1.1.19), agmatine iminohydrolase (AIH; EC 3.4.3.12), and *N*-carbamoylputrescine amidohydrolase (CPA; EC 3.5.1.53). Spd and Spm are the result of sequential additions of aminopropyl moieties to Put and Spd by the enzymes Spd synthase (SPDS; EC 2.5.1.16) and Spm synthase (SPMS; EC 2.5.1.22), respectively. Decarboxylated S-adenosylmethionine (dcSAM) is used by both enzymes as donor molecule of aminopropyl groups, and is synthesized from the decarboxylation of S-adenosylmethionine (SAM) in a reaction catalyzed by SAM decarboxylase (SAMDC; EC 4.1.1.50). Spd and dcSAM can also form a structural isomer of Spm, known as thermospermine (tSpm), in a reaction catalyzed by tSpm synthase (tSPMS; EC 2.5.1.79). Characterization of the Arabidopsis genome has allowed to identify two genes encoding ADC (*ADC1* and *ADC2*) (Watson and Malmberg, 1996; Watson et al., 1997) and one for each AIH and CPA (Janowitz et al., 2003; Piotrowski et al., 2003). The Arabidopsis genome also carries two genes encoding SPDS (*SPDS1* and *SPDS2*) (Hanzawa et al., 2002), one coding for SPMS (*SPMS)* (Panicot et al., 2002), another one coding for tSPMS (*ACL5*) (Knott et al., 2007; Kakehi et al., 2008), and at least four coding for SAMDC (*SAMDC1-4*) (Urano et al., 2003).

PAs are catabolized through the activity of diamine oxidases (DAO; EC 1.4.3.6) and polyamine oxidases (PAO; EC 1.5.3.3). DAOs display high affinity for diamines, like Put, producing Δ^1^-pyrroline, hydrogen peroxide (H_2_O_2_) and ammonia, while PAOs oxidize secondary amine groups from Spd and Spm leading to the formation of 4-aminobutanal or (3-aminopropyl)-4-aminobutanal, along with 1,3-diaminopropane (DAP) and H_2_O_2_. PAOs are also able to catalyze the back-conversion of Spm to Spd, producing 3-aminopropanal and H_2_O_2_. Some PAO isoforms are also involved in back-conversion processes of tSpm to Spm, and Spm to Put, that lead to the production of H_2_O_2_ (Moschou et al., 2012). At least five genes encoding putative PAOs (Alcazar et al., 2006; Takahashi et al., 2010) and 10 genes encoding putative DAOs (Planas-Portell et al., 2013) are present in the Arabidopsis genome.

PA metabolism is altered in a variety of plant hosts in response to several pathogens (Walters, 2003a,b), suggesting a role for PAs in the biotic defense response. However, the precise mechanism(s) of action by which PAs could exert this defensive role remains unclear, although some possible mechanisms of action have been proposed. An up-regulation of PA biosynthesis and catabolism has been observed during hypersensitive response (HR) induced by the powdery mildew fungus *Blumeria graminis* f. sp. *hordei* in barley (Cowley and Walters, 2002), as well as in tobacco plants exposed to tobacco mosaic virus (TMV) (Marini et al., 2001). PA catabolism produces H_2_O_2_, a reactive oxygen species (ROS) that could have an antimicrobial effect as well as participate in host defense mechanisms, including cell wall modifications, or act as a signal molecule triggering processes like HR (Walters, 2003a). PA oxidation has also been observed in compatible interactions between plant hosts and different types of pathogens. Spm accumulates in the apoplast of tobacco plants infected by *Pseudomonas syringae* pv. *tabaci*, and its oxidation by PAO leads to the production of H_2_O_2_ (Moschou et al., 2009). Moreover, PAO overexpression enhances tobacco tolerance to biotrophic bacteria *P. syringae* as well as to the hemibiotrophic oomycete *Phytophtora parasitica* var *nicotianae* (Moschou et al., 2009). Apoplastic PA accumulation and further oxidation has also been observed in tobacco leaves exposed to the biotrophic bacterium *Pseudomonas viridiflava* (Burkholder) Dowson, restricting bacterial growth in the host (Marina et al., 2008).

A role for Spm in defense signaling has been pointed out in *A. thaliana*, in which exogenous Spm induces a set of genes that are also expressed in response to the cucumber mosaic virus (CMV) infection (Mitsuya et al., 2009). Changes in Spm metabolism and subcellular localization have been associated with plant host responses to pathogenic attack. Induction of acidic pathogenesis-related proteins (PR) observed during TMV infection in tobacco is produced by Spm accumulation in the leaf apoplast, and this induction is not dependent on Salicylic acid (SA) (Yamakawa et al., 1998). Moreover, Spm accumulation, which can also be mimicked by exogenous Spm application on tobacco leaves, triggers a “Spm-signaling pathway” that causes mitochondrial dysfunction by activation of mitogen-activated protein kinases and increase of the expression of a set of HR-specific genes, leading tobacco leaf cells to develop defense responses and HR-like cell death (Takahashi et al., 2003, 2004; Mitsuya et al., 2007).

More recently, the manipulation of PA levels by transgenic approaches and the use of loss or gain-of-function mutations have emerged as new tools to gain knowledge about the role of PAs in plant stress responses (Alcázar et al., 2010; Gill and Tuteja, 2010). Consistent with the hypothesis that Spm could perform a key role in defense signaling, transgenic 35S::*SPMS* Arabidopsis plants accumulate Spm and are more resistant to infection by *P. viridiflava* than the wild type (WT). On the contrary, Spm-deficient *spms* mutant lines are more susceptible to infection (Gonzalez et al., 2011). Comparison of the transcriptomes of Spm-accumulating and Spm-deficient mutants showed that many genes only overexpressed in 35S::*SPMS* lines participate in pathogen perception and defense responses, including several families of disease resistance genes, transcription factors, kinases, and nucleotide- and DNA/RNA-binding proteins (Gonzalez et al., 2011). At the same time, most of those genes appear also induced in other Spm–accumulating lines obtained by overexpression of *SAMDC1* gene (Marco et al., 2011).

In this work we have compared the expression levels of some disease resistance genes between *SAMDC1*-overexpressing lines and WT plants. Furthermore, we have also compared the effect of Spm accumulation on the susceptibility to the bacteria *P. syringae*, and to the oomycete *Hyaloperonospora arabidopsidis*. In both cases, *SAMDC1*-overexpressing lines were more resistant to infection than WT plants.

## Materials and methods

### Plant growth conditions

Infection by the bacterial pathogen *P. syringae* and by the oomycete *H. arabidopsidis* was tested in three Arabidopsis transgenic lines (pBISDCs-S3', pBISDCs-S9', pBISDCs-S15) overexpressing the *SAMDC1* gene under the control of *CaMV35S* constitutive promoter (Marco et al., 2011) and in the ecotype Col-0, WT obtained from the Nottingham Arabidopsis Stock Center (University of Nottingham, Loughborough, UK). pBISDCs lines exhibit a similar phenotype to WT plants in terms of growth and development.

Arabidopsis seeds were sown in pots with a 1:1:1 mixture of soil, vermiculite and sand, stratified for 2 days at 4°C, and transferred to growth chambers. Plants were grown in Sanyo MLR-350 (Sanyo Electric Co., Japan) chambers, either under long day conditions (illumination at 23°C for 16 h, darkness at 16°C for 8 h), or short day conditions (illumination at 18°C for 10 h, darkness at 16°C for 14 h), and watered with mild nutrient solution (recipe from Arabidopsis Biological Resource Center, The Ohio State University, USA, handling plants and seeds guide, http://www.biosci.ohio-state.edu/pcmb/Facilities/abrc/handling.htm).

Seedlings were also grown on plates under long day conditions. Seeds surface was sterilized by washing in 30% (v/v) commercial bleach, 0.01% (v/v) Triton X-100 by 10 min and rinsed three times with sterile distilled water. Sterile seeds were plated on 4% agar plates containing one half strength MS medium (½MS) (Murashige and Skoog, 1962). When required, seedlings were also grown in ½MS plates supplemented with 0.1, 0.5, and 1 mM of Put, Spd, or Spm. Whole seedling samples were taken after 5 days of growth and immediately frozen in liquid Nitrogen and stored at −80°C for RNA extraction.

### *P. syringae* infection conditions and disease evaluation

*P. syringae* pv. *maculicola* ES4326 and pv. *tomato* DC3000, kindly supplied by Dr. Jürgen Zeier (Julius-von-Sachs-Institute of Biological Sciences, University of Würzburg, Germany) and Dr. John Stavrinides (Department of Botany, University of Toronto, Canada), respectively, were used to infect Arabidopsis WT plants, as well as pBISDCs *SAMDC1*-overexpressing lines. Both bacterial strains were cultivated at 28°C in King's B medium (King et al., 1954). Streptomycin (100 μg/ml) or rifampicin (50 μg/ml) were added to select growth of strains pv. *maculicola* ES4326 and pv. *tomato* DC3000, respectively. For plant inoculation, bacterial cells were grown until cultures reached an OD_600_ of 0.1, collected by centrifugation, washed, and resuspended in 10 mM MgCl_2_ to a final concentration of 3 × 10^6^ CFU/ml.

Leaves of 15 day-old plants grown in long day conditions were inoculated with *P. syringae* according to Zeier et al. (2004). Briefly, bacterial suspension was inoculated on the abaxial surface of leaves, using a 1-mL syringe without a needle. Control inoculations were performed with 10 mM MgCl_2_ pH 7.0. 10 different plants were inoculated with each bacterial strain and disease extension was quantified by two approaches. Image analysis was used to evaluate chlorosis in the diseased plants. Leaves were scanned at 600 dpi and images were processed using the *Image Processing Tool Kit 5.0* (Reindeer Graphics Inc.) and *Photoshop 7.0* softwares (Adobe Systems, Inc) to measure leaf area. Since yellow generates a lighter shade of gray than green, chlorosis-induced yellowing was estimated by the conversion of leaf images to gray tones and the determination of their luminance percentages. On the other hand, severity of infection was also estimated by measuring *in planta* bacterial growth. Foliar extracts were obtained by cutting three leaf discs from each plant with a 0.5 cm-diameter borer, homogenizing them with 10 mM MgCl_2_. Then, serial dilutions of the extracts thus obtained were plated on King's B agar medium supplemented with the appropriate antibiotic. The number of colony forming units (CFU) was determined after 24 h incubation at 28°C. Both, images and plant extracts were obtained 2 and 3 days after infection. Samples were also harvested, frozen in liquid Nitrogen and stored at −80°C for RNA extraction.

### *H. arabidopsidis* infection conditions and disease evaluation

*H. arabidopsidis* isolate *Noks1*, was used to infect Col-0 WT Arabidopsis plants by using a dry powder containing pathogen oospores, obtained from previously infected leaves kindly supplied by Dr. Mahmut Tör (Warwick HRI, University of Warwick, UK). Methods for subculturing *H. arabidopsidis* and preparing inoculum for experiments were modified from Tör et al. (2002). Powder was sprinkled on pots containing Arabidopsis seeds, which were then stratified and left to germinate under short day conditions as described above. Presence of sporangiophores on cotyledons was checked daily and cotyledons with abundant sporulation were selected. *H. arabidopsidis* conidiospores were released from infected cotyledons by rinsing infected tissues with distilled water followed by centrifugation. Sedimented conidiospores were resuspended in distilled water, counted with a Neubauer chamber (Hauser Scientific Partnership, HORSHAM, PA 19044) and diluted to a final concentration of 5 × 10^4^ conidiospores/ml.

Inocula consisting of 2 μl of this conidiospores suspension were applied on cotyledons of 14 day-old WT plants and pBISDCs transgenic lines. After inoculation, plants were grown under short day conditions and periodically sprinkled with water to maintain moisture. A control batch of plants was inoculated with distilled water and grown in parallel. 10 days after inoculation, 25 leaves were sampled for each line and the developed sporangiophores were counted.

### RNA extraction

Total RNA was extracted from plant tissues using Total Quick RNA Cells and Tissues Kit (Talent SRL, Italy), following the protocol established by manufacturer. RNA was quantified by measuring the absorbance at 260 nm, and their integrity was checked by denaturing agarose gel electrophoresis.

### Quantitative RT-PCR

RNA was treated with RNase free-DNAse (Roche diagnostics, Spain) to remove contaminating genomic DNA. A total of 1 μg of DNA free-total RNA was reverse transcribed to first-strand complementary DNA (cDNA) with random hexamers using SuperScript®III First-Strand Synthesis System 1st (Invitrogen, Spain) according to manufacturer's instructions. Quantitative real time PCR (qRT-PCR) was performed on GeneAmp®5700 Sequence Detection System (PE Applied Biosystems, Japan), using Power SYBR®Green PCR Master Mix (PE Applied Biosystems). 20 μl reactions contained 1 μl of cDNA, 100 nM of each pair of target primers (FW and REV) and 10 μl of SYBR Green PCR Master Mix. PCR conditions were as follows: 95°C for 10 min, followed by 40 cycles of 95°C for 30 s and 60°C for 1 min. Three technical replicates from three independent biological experiments were performed for qRT-PCR analyses. Primers used for real-time PCR are described in Table 1. The efficiency of primers and the data were analyzed according to the 2^−ΔΔCT^ method (Livak and Schmittgen, 2001). Gene coding for actin-2 (*ACT-2*; *AT3G18780*; An et al., 1996) was used as a reference gene.

**Table 1:** **Primers used in qRT-PCR analyses**.

**Gene**	**AGI locus**	**Forward (5′–3′)**	**Reverse (5′–3′)**
*PR-1*	AT2G14610	CCACAAGATTATCTAAGGGGTC	TTCCACTGCATGGGACCTA
*PR-2*	AT3G57260	CATCCTCGACGTTCCCAGTT	TGTCGGCCTCCGTTTGA
*PR-5*	AT1G75050	AACGGCGGCGGAGTTC	GCCGCCATCGCCTACTAGA
*CYP79F1*	AT1G16410	CATCCGTGCCATCACCATAA	CAAATCTGCGTCCCGCTCTCT
*WAK1*	AT1G21245	TGCTCTCAGGTCAAAAGGCATT	CGCAAAGTAACTCACCAGATGT
*FLS2*	AT5G46330	CCTGGACCTGTCTCACAACCA	ACGTAAGATTCATCCTTCCGAA
*LOX2*	AT3G45140	CAACGACAACAAGGATAAGA	CTGGCGACTCATAGAACT
*AOC1*	AT3G25760	CGTCCCATTCACAAACAACTC	CAGAGACCAGCCGTGATTCC
*AOC2*	AT3G25780	ACTGGAACGGCGGTTACG	GGCTCCATCGCCTTAGCTT
*AOS*	AT5G42650	CGGGCGGGTCATCAAG	GCCGTTGGATTTAATCACAGAT
*DAD1*	AT2G44810	GGAGACGCCGTGGGTTT	GGCGAGTCACGGCTCA
*JMT*	AT1G19640	CCAACATCACTTACTATATTCAT	GAAGAACTCGCATTACCT
*ACT-2*	AT3G18780	GATTCAGATGCCCAGAAAGTCTTG	TGGATTCCAGCAGCTTCCAT

### Statistical analyses

Data was analyzed by One-Way Analysis of Variance (ANOVA) followed by *post-hoc* comparisons by Tukey's HSD or Dunnet's T3 *t*-test. A probability level < 0.05 was considered statistically significant. Calculations were performed using IBM®SPSS®Statistics v20.0 Software.

## Results

### pBISDCs transgenic lines show constitutive elevated expression levels of disease response and jasmonic acid metabolism genes

**Figure 1:**
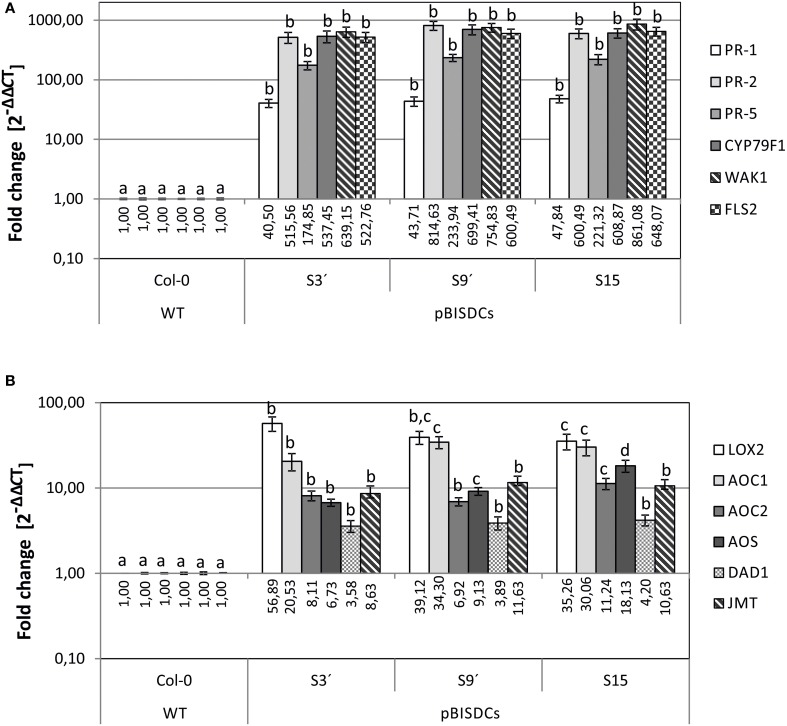
**qRT-PCR analysis of biotic stress-related genes in leaves of 4-week old *A. thaliana* WT and pBISDCs transgenic lines overexpressing *SAMDC1* (S3', S9, and S15)**. Expression levels were determined for a set of biotic stress defense-related genes **(A)**, as well as jasmonate and methyl-jasmonate biosynthesis genes **(B)**. For each gene, data is expressed as fold change relative to the level measured in WT plants (2^−ΔΔCT^). Graph show the mean of three biological replicates ± standard deviation. Significant differences between plant lines are indicated with letters (ANOVA, Tukey HSD test, *p* < 0.05).

Previous comparison of the transcriptomes of WT and pBISDCs transgenic lines showed that overexpression of *SAMDC1* gene in Arabidopsis leads to higher Spm levels and to the induction of a set of genes enriched in functional categories involved in defense-related processes against both biotic and abiotic stresses (Marco et al., 2011). Some of these genes were selected and their expression checked by qRT-PCR to confirm the results obtained in transcriptome studies. Expression levels of genes that encode for pathogenesis-related proteins *PR-1* (*AT2G14610*), *PR-2* (*AT3G57260*) and *PR-5* (*AT3G57260*) (Uknes et al., 1992; Van Loon et al., 2006); *CYP79F1* (*AT1G16410*), a cytochrome P450 involved in the biosynthesis of aliphatic glucosinolates (Hansen et al., 2001; Chen et al., 2003); Cell-Wall associated kinase *WAK1* (*AT1G21245*; Verica and He, 2002) as well as the flagellin receptor *FLS2* (*AT5G46330*; Gomez-Gomez and Boller, 2000) were determined by qRT-PCR (Figure 1A). Our results demonstrate that all disease response genes tested showed a dramatic increase in mRNA abundance in transgenic lines overexpressing *SAMDC1*. The expression level of these genes was very similar in all the pBISDCs lines selected, ranging from 100 to 800-fold higher than WT, except for *PR-1*, whose expression increased less than 100-fold (Figure 1A).

Previous transcriptome analysis showed that another set of overexpressed genes in *SAMDC1-*overexpressing lines were enriched in the category of genes related to jasmonic acid biosynthesis and response (Marco et al., 2011). Expression levels of genes that encode jasmonate (JA) and methyljasmonate (MeJA) biosynthesis enzymes such as Chloroplast lipoxygenase *LOX2* (*AT3G45140*; Bell et al., 1995); Allene oxide cyclase, *AOC1* (*AT3G25760*) and *AOC2* (*AT3G25780*) (Stenzel et al., 2003); Allene oxide synthase *AOS* (*AT5G42650*; Kubigsteltig et al., 1999); chloroplastic phospholipase A1 *DAD1* (*AT2G44810*; Ishiguro et al., 2001), and jasmonate O-methyltransferase *JMT* (*AT1G19640*; Seo et al., 2001), were also tested by qRT-PCR in WT and pBISDCs lines (Figure 1B). Higher levels of expression were observed for all JA and MeJA biosynthesis genes in *SAMDC1*-overexpressing lines when compared to WT plants, with *LOX2* showing the most pronounced increase, with a mean of 43.76-fold higher than WT (Figure 1B).

### Spm treatment raises the expression of defense-related genes *PR-1, PR-5*, as well as jasmonic acid biosynthesis *AOS* and *AOC1* genes

**Figure 2:**
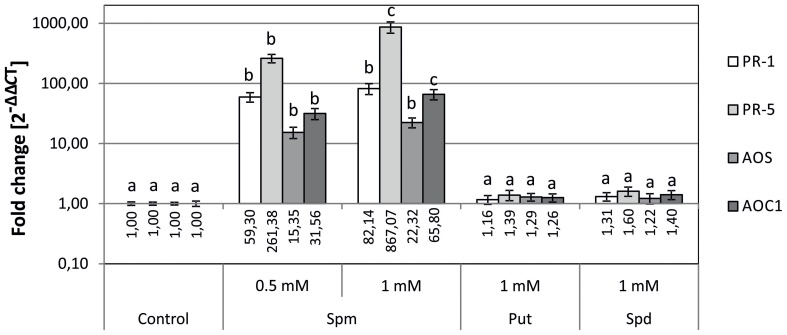
**Effects of external polyamine treatment on the expression of biotic stress defense-related genes (*PR-1* and *PR5*) and jasmonate biosynthesis genes (*AOS* and *AOC1*)**. Plants were grown for 5 days in plates supplemented with Put, Spd, or Spm, as well as in control plates without the amendment of PAs. Expression levels were determined by qRT-PCR. For each gene, data is expressed as fold change relative to the level measured in WT plants in control conditions (2^−ΔΔCT^). Graph show the mean of three biological replicates ± standard deviation. Significant differences between treatments are indicated with letters (ANOVA, Tukey HSD test, *p* < 0.05).

To determine whether the changes in gene expression observed in *SAMDC1*-overexpressing plants (Figure 1) were due to their higher Spm levels than WT plants (Marco et al., 2011), the effect of exogenous applied PAs on expression of defense response and JA biosynthesis genes was tested. WT seeds were sown on MS media containing exogenously supplied PAs and expression levels of *PR-1*, *PR-5*, *AOS* and *AOC1* genes were determined by qRT-PCR 5 days after germination. A positive correlation between exogenous Spm concentration and transcript level was observed for the set of defense-related and JA biosynthesis genes analyzed (Figure 2). Conversely, exogenous addition of Put or Spd did not produce significant changes in gene expression (Figure 2).

### Overexpression of *SAMDC1* enhances resistance to the oomycete *H. arabidopsidis* and to the bacteria *P. syringae*

The qRT-PCR analyses shown above (Figure 1), as well as previous transcriptome analysis of pBISDCs plants (Marco et al., 2011), suggested that *SAMDC1*-overexpressing plants, with elevated Spm levels, have constitutively activated a set of genes related to the defense response of plants to pathogenic microorganisms. Therefore, the response of the transgenic and WT lines against infection by bacterial or oomycete pathogens was studied.

**Figure 3:**
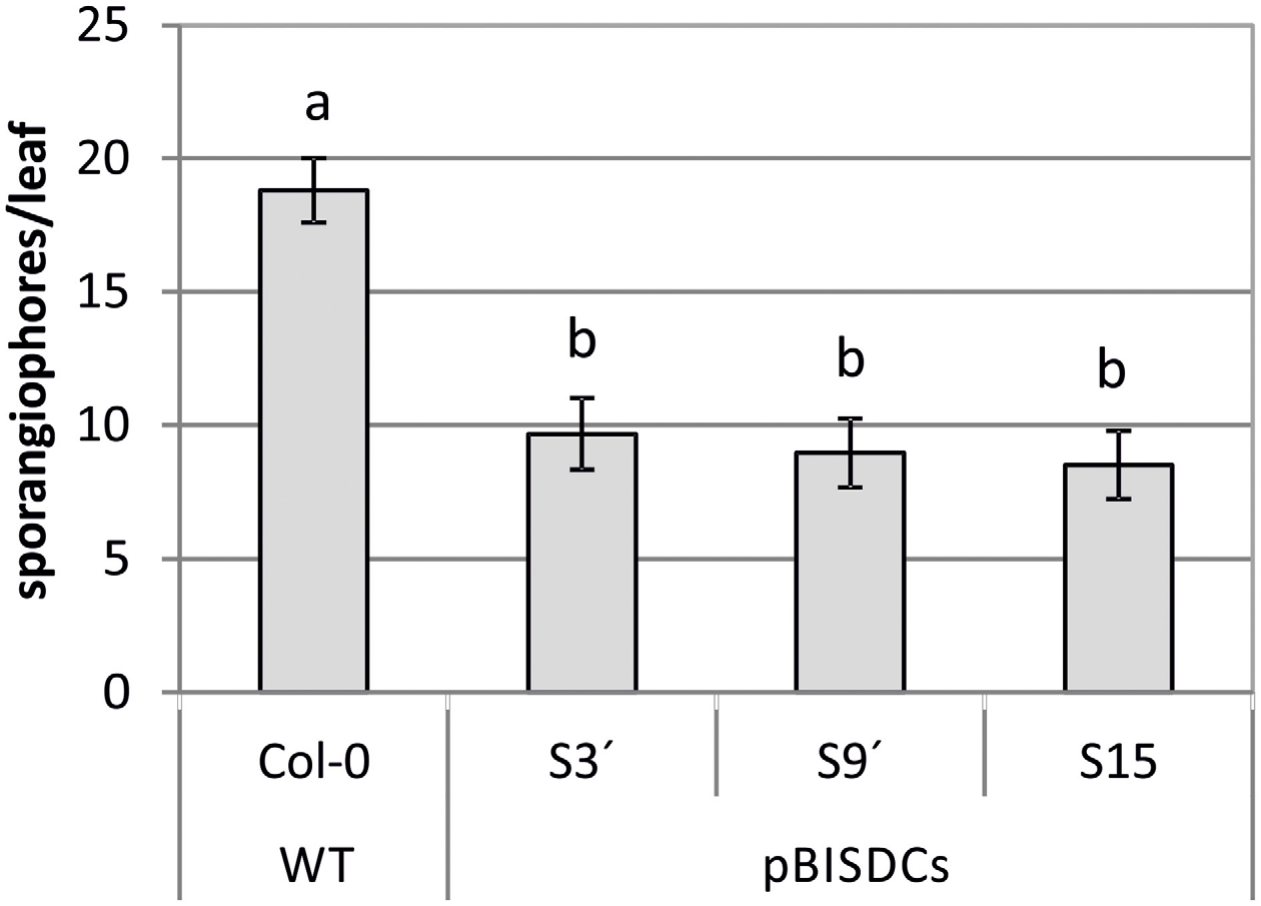
**Effects of *H. arabidopsidis* infection in Arabidopsis WT and pBISDCs transgenic lines overexpressing *SAMDC1* (S3', S9', and S15)**. Fourteen-day old plants were inoculated with a conidiosporium suspension of *H. arabidopsidis* isolate *Noks1*. Disease extension was estimated 10 days after inoculation by counting the number of sporangiophores developed in each leaf. Twenty-five leaves were sampled for each line. Graph shows the mean ± standard deviation. Significant differences between plant lines are indicated with letters (ANOVA, Tukey HSD test, *p* < 0.05).

WT and PBISDCs lines were infected with *H. arabidopsidis* isolate *Noks1*. Two-week-old plants were exposed to a suspension containing *H. arabidopsidis* conidiospores and extension of infection was evaluated after 10 days by counting the number of sporangiophores by leaf. The number of sporangiophores by leaf observed in pBISDCs lines was approximately a half of the number obtained in WT plants, suggesting a lower propagation of *H. arabidopsidis* (Figure 3).

**Figure 4:**
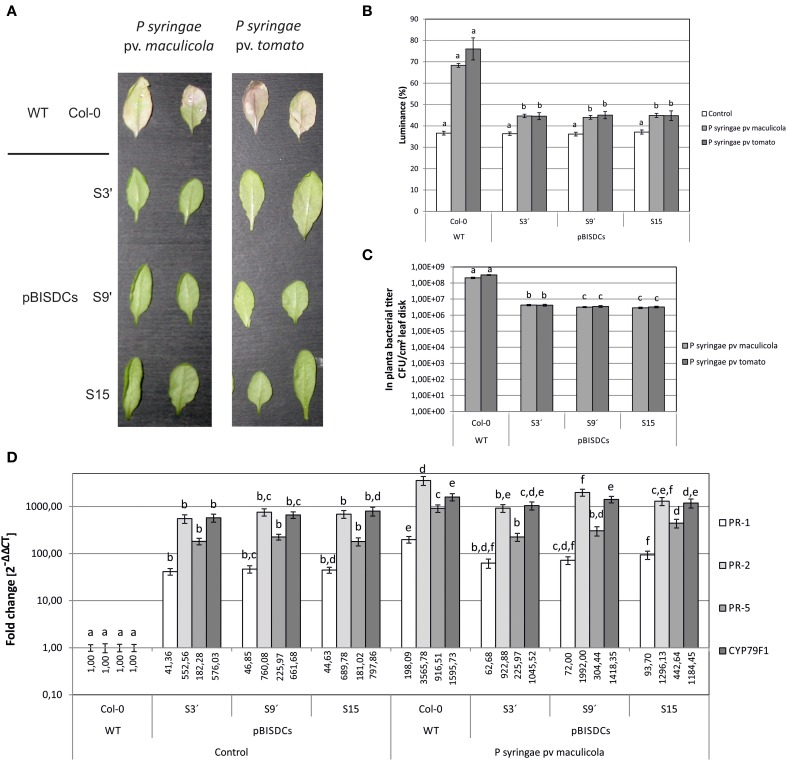
**Effects of *P. syringae* infection in Arabidopsis WT and pBISDCs transgenic lines overexpressing *SAMDC1* (S3', S9, and S15)**. Leaves of 15 day-old plants were inoculated with a suspension of *P. syringae* strains pv. *maculicola* ES4326 or pv. *tomato* DC3000. Ten different plants for each line were inoculated. Disease extension was evaluated for each strain 3 days after inoculation visually **(A)**, as well as by quantifying leaf luminance percentage by image analysis using the *Image Processing Tool Kit 5.0* and *Photoshop 7.0* softwares **(B)**. Also, propagation of *P. syringae in planta* was estimated by determination of the number of CFU/cm^2^ of leaf disk **(C)**. Ten different plants for each line were inoculated. Results show the mean ± standard deviation obtained for each combination of line and bacterial strain. For each condition, significant differences between lines are indicated with letters [ANOVA, Tukey HSD test (luminance data) or Dunnet's T3 test (CFU data), *p* < 0.05]. Plant response to *P. syringae* pv. *maculicola* infection was also studied by comparison of the expression levels for a set of biotic stress defense-related genes 3 days after inoculation with the bacterial strain or control inoculums **(D)**. For each gene, data is expressed as fold change relative to the level measured in WT plants inoculated with control inoculum (2^−ΔΔCT^). Graph show the mean of three biological replicates ± standard deviation. Significant differences between plant lines are indicated with letters (ANOVA, Tukey HSD test, *p* < 0.05).

Additionally, two strains of the bacterial pathogen *P. syringae*, pv. *maculicola* ES4326 and pv. *tomato* DC3000, were also inoculated in 2-week old leaves of WT and pBISDCs transgenic lines. Both strains infect Arabidopsis leaves and cause initial chlorosis followed by appearance of dark spots. Progress of infection was followed by visual estimation of chlorosis appearance (Figure 4A) or by quantifying leaf luminance percentage after conversion to grayscale and image analysis (Figure 4B). Three days after inoculation, visual symptoms of chlorosis were observed in WT plants inoculated with either *P. syringae* strains (Figure 4A) along with an increase in leaf luminance (Figure 4B). However, *SAMDC1*-overexpressing lines did not show yellowing symptoms 3 days after inoculation (Figure 4A), with luminance levels close to control conditions (Figure 4B), suggesting at least a delay in infection. *P. syringae* propagation *in planta* was also estimated by the determination of CFU in foliar disks 3 days after inoculation. Compared to WT plants, pBISDCs lines showed a 10-fold reduction in the propagation of both strains of *P. syringae* (Figure 4C).

In addition, leaves of plants of each line infected with *P. syringae* pv. *maculicola* or exposed to control inoculum were collected 3 days after infection and expression of defense-related genes *PR-1*, *PR-5*, *CYP79F1*, and *WAK1* was determined by qRT-PCR (Figure 4D). A dramatic induction of the expression of the four genes was observed in WT leaves, ranging from 200-fold in the case of *PR-1* or 1000 to 2000-fold in the case of *PR-2*, *PR-5* or *CYP79F1*. In turn, the expression levels of those genes in uninfected leaves of *SAMDC-1* transgenic plants were higher than in uninfected WT plants (Figure 4D) and similar to the levels reached in them after infection (Figure 4D).

## Discussion

Previous studies have shown that overexpression of *SAMDC1* or *SPMS* genes in Arabidopsis leads to plants with higher Spm content than WT plants (Gonzalez et al., 2011; Marco et al., 2011). Transcriptome studies have pointed out that a common set of 233 genes is induced in those Spm-accumulating lines. This set of genes is enriched in functional categories involved in defense-related processes during both biotic and abiotic stresses as well as JA biosynthesis and response (Marco et al., 2011). These results suggest a connection between the increase of Spm levels and the induction of biotic stress responses. The connection among Spm levels and biotic stress responses is confirmed when the set of induced genes found at *SAMDC1*-overexpressing lines is compared to a set of 312 ESTs differentially expressed during Systemic Acquired Resistance (SAR) in Arabidopsis (Maleck et al., 2000). When this comparison is made, a set of 71 common genes emerges, including transcripts coding for pathogenesis-related proteins PR-1, PR-2 as well as the JA biosynthesis enzyme LOX2, which appear overexpressed in both Arabidopsis SAR and pBISDCs transcriptomes (Maleck et al., 2000; Marco et al., 2011).

The gene expression analysis made in the present work confirmed that expression levels of *PR-1*, *PR-2*, and *PR-5* genes, are higher in Spm-accumulating pBISDCs lines than in WT plants (Figure 1A), as previously observed by transcriptome studies (Marco et al., 2011). Exogenously applied Spm also produces *PR-1* and *PR-5* induction in WT plants (Figure 2), suggesting that their expression levels in pBISDCs lines could be related to the higher Spm levels found in these plants. As expected, those genes are also induced when WT plants are infected by *P. syringae* pv. *maculicola* (Figure 4D). Induction of acidic PR proteins in response to abiotic stress and Spm treatment has been previously described in tobacco (Yamakawa et al., 1998). Moreover, *PR-1* induction by Spm in Arabidopsis has been also reported by Mitsuya et al. (2009). Additionally, overexpression of *SPDS* in *Citrus sinensis Osbeck* (sweet orange) leads to plants with higher Spm levels, as well as to the overexpression of genes that code for putative PR proteins, like *PR-4A* and *PR-10A* (Fu et al., 2011; Fu and Liu, 2013).

Our qRT-PCR analysis also confirmed the up-regulation of the defense-related genes *CYP79F1*, *WAK1* and *FLS2* in *SAMDC1*-overexpressing lines (Figure 1A). Selection of these genes was made based on previous transcriptome data (Marco et al., 2011) and the different roles played by them during plant pathogenic responses. CYP79F1, a member of the cytochrome P450 (CYP) superfamily, is a key enzyme in the biosynthesis pathway of aliphatic glucosinolates from methionine (Hansen et al., 2001; Chen et al., 2003). Glucosinolates constitute an essential part of plant defense secondary metabolites (Halkier and Gershenzon, 2006). Diverse defense pathways control glucosinolate biosynthesis by activation of different subsets of biosynthetic enzymes (CYP among them), leading to the accumulation of specific glucosinolate profiles (Mikkelsen et al., 2003). In this trend, *CYP79F1* is induced by MeJA (Mikkelsen et al., 2003; Guo et al., 2013) and our results demonstrate that it is also induced by *P. syringae* pv. *maculicola* infection (Figure 4D). WAK1 is the most studied member of a five-member family of Cell-Wall associated protein kinases (WAK1-5) (Verica and He, 2002). *WAK1* is induced by *P. syringae* pv. *maculicola* infection and by SA treatment (Schenk et al., 2000). Expression of *WAK1* is required by the plant to survive against lethal accumulation of SA during plant–pathogen interactions (He et al., 1998), and its ectopic overexpression confers resistance to *Botrytis cinerea* (Brutus et al., 2010). *WAK1* is also induced by MeJA and ethylene (Schenk et al., 2000). In addition, another member of WAK family, *WAK2*, appears up-regulated in pBISDCs and Arabidopsis SAR transcriptomes (Maleck et al., 2000; Marco et al., 2011). FLS2 is a receptor kinase essential in the perception of flagellin, a potent elicitor of the defense response to bacterial infection (Gomez-Gomez and Boller, 2000). Flagellin perception initiates a battery of downstream defense pathways that leads to stomatal closure to avoid bacterial invasion, as well as to the activation of mechanisms inhibiting bacterial multiplication in the plant apoplast (Zipfel et al., 2004; Melotto et al., 2006).

Furthermore, levels of expression of genes coding for JA and MeJA biosynthesis enzymes, including LOX2, were also checked by qRT-PCR (Figures 1B, 2). Again, Spm was the unique PA able to induce *AOS* and *AOC1* genes by external treatment (Figure 2), suggesting that the induction of JA and MeJA biosynthesis genes observed in *SAMDC1*-overexpressing plants (Figure 1B) could be promoted by their modified Spm levels. This induction could lead to the rise of JA levels in pBISDCs plants and promote JA-mediated defense mechanisms. It has been previously described that Spm treatment induces JA biosynthesis in lima bean, promoting the production of herbivore-induced volatile terpenoids that attract predatory mites (Ozawa et al., 2009). Previous studies have suggested possible interactions between PAs and JA in disease response. MeJA treatment increases PA levels and renders an improved disease response in barley seedlings exposed to powdery mildew (Walters et al., 2002), as well as in wheat plants infected with leaf rust (Haggag and Abd-El-Kareem, 2009) or in loquat fruits inoculated with *Colletotrichum acutatum* spores (Cao et al., 2014).

*SAMDC1*-ovexexpressing plants show an enhanced tolerance when infected by any of the two strains of *P. syringae* assayed (Figure 4A) and by the oomycete *H. arabidopsidis* (Figure 3), in terms of *in planta* pathogen propagation. Enhanced tolerance to biotrophic bacteria *P. viridiflava* has also been observed in Arabidopsis Spm-accumulating lines obtained by overexpression of *SPMS*, as well as in WT plants treated with exogenous Spm (Gonzalez et al., 2011). In addition, sweet orange *SPDS*-overexpressing plants are also less susceptible to *Xanthomonas axonopodis* pv. *citri*, the bacterial agent that causes citrus canker (Fu et al., 2011).

In summary, qRT-PCR studies conducted in this work confirmed that pBISDCs lines have an up-regulated expression of genes that code for members of the pathogen defense system, as suggested by previous transcriptome studies (Marco et al., 2011). This constitutive activation of the defense-response mechanisms has also a positive impact on the susceptibility of pBISDCs lines against bacterial (Figure 4) and oomycete infection (Figure 3). Results obtained in this study add more evidence to the role of Spm in plant response to biotic stress, and reinforce the hypothesis that, among the different mechanisms postulated by which Spm could exert their protective action, transcriptional changes of defense genes might play an important role. It remains to be determined which of the changes in gene expression observed in the transcriptome of Spm accumulating plants are the result of the direct action of Spm or the consequence of intricate cross-talking between Spm and other biotic defense-signaling pathways, including JA and MeJA.

### Conflict of interest statement

The authors declare that the research was conducted in the absence of any commercial or financial relationships that could be construed as a potential conflict of interest.

## References

[B1] Alcázar R. Altabella T. Marco F. Bortolotti C. Reymond M. Koncz C. (2010). Polyamines: molecules with regulatory functions in plant abiotic stress tolerance. Planta 231, 1237–1249 10.1007/s00425-010-1130-0 20221631

[B2] Alcazar R. Marco F. Cuevas J. C. Patron M. Ferrando A. Carrasco P. (2006). Involvement of polyamines in plant response to abiotic stress. Biotechnol. Lett. 28, 1867–1876 10.1007/s10529-006-9179-3 17028780

[B3] An Y. Q. McDowell J. M. Huang S. McKinney E. C. Chambliss S. Meagher R. B. (1996). Strong, constitutive expression of the Arabidopsis ACT2/ACT8 actin subclass in vegetative tissues. Plant J. 10, 107–121 10.1046/j.1365-313X.1996.10010107.x 8758981

[B4] Bell E. Creelman R. A. Mullet J. E. (1995). A chloroplast lipoxygenase is required for wound-induced jasmonic acid accumulation in Arabidopsis. Proc. Natl. Acad. Sci. U.S.A. 92, 8675–8679 10.1073/pnas.92.19.8675 7567995 PMC41029

[B5] Brutus A. Sicilia F. Macone A. Cervone F. De Lorenzo G. (2010). A domain swap approach reveals a role of the plant wall-associated kinase 1 (WAK1) as a receptor of oligogalacturonides. Proc. Natl. Acad. Sci. U.S.A. 107, 9452–9457 10.1073/pnas.1000675107 20439716 PMC2889104

[B6] Cao S. Cai Y. Yang Z. Joyce D. C. Zheng Y. (2014). Effect of MeJA treatment on polyamine, energy status and anthracnose rot of loquat fruit. Food Chem. 145, 86–89 10.1016/j.foodchem.2013.08.019 24128452

[B7] Chen S. Glawischnig E. Jorgensen K. Naur P. Jorgensen B. Olsen C. E. (2003). CYP79F1 and CYP79F2 have distinct functions in the biosynthesis of aliphatic glucosinolates in Arabidopsis. Plant J. 33, 923–937 10.1046/j.1365-313X.2003.01679.x 12609033

[B8] Cowley T. Walters D. R. (2002). Polyamine metabolism in barley reacting hypersensitively to the powdery mildew fungus *Blumeria graminis* f. sp. hordei. Plant Cell Environ. 25, 461–468 10.1046/j.0016-8025.2001.00819.x

[B9] Fu X. Z. Chen C. W. Wang Y. Liu J. H. Moriguchi T. (2011). Ectopic expression of MdSPDS1 in sweet orange (*Citrus sinensis* Osbeck) reduces canker susceptibility: involvement of H(2)O(2) production and transcriptional alteration. BMC Plant Biol. 11:55 10.1186/1471-2229-11-55 21439092 PMC3078878

[B10] Fu X. Z. Liu J. H. (2013). Transcriptional profiling of canker-resistant transgenic sweet orange (*Citrus sinensis* Osbeck) constitutively overexpressing a spermidine synthase gene. Biomed. Res. Int. 2013:918136 10.1155/2013/918136 23509803 PMC3591164

[B11] Gill S. Tuteja N. (2010). Polyamines and abiotic stress tolerance in plants. Plant Signal. Behav. 5, 26–33 10.4161/psb.5.1.10291 20592804 PMC2835953

[B12] Gomez-Gomez L. Boller T. (2000). FLS2: an LRR receptor-like kinase involved in the perception of the bacterial elicitor flagellin in Arabidopsis. Mol. Cell 5, 1003–1011 10.1016/S1097-2765(00)80265-8 10911994

[B13] Gonzalez M. E. Marco F. Minguet E. G. Carrasco Sorli P. Blázquez M. A. Carbonell J. (2011). Perturbation of spermine synthase gene expression and transcript profiling provide new insights on the role of the tetraamine spermine in *Arabidopsis thaliana* defense against *Pseudomonas viridiflava*. Plant Physiol. 156, 2266–2277 10.1104/pp.110.171413 21628628 PMC3149955

[B14] Guo R. Shen W. Qian H. Zhang M. Liu L. Wang Q. (2013). Jasmonic acid and glucose synergistically modulate the accumulation of glucosinolates in *Arabidopsis thaliana*. J. Exp. Bot. 64, 5707–5719 10.1093/jxb/ert348 24151308 PMC3871825

[B15] Haggag W. Abd-El-Kareem F. (2009). Methyl jasmonate stimulates polyamines biosynthesis and resistance against leaf rust in wheat plants. Arch. Phytopathol. Plant Protect. 41, 16–31 10.1080/03235400600914355

[B16] Halkier B. A. Gershenzon J. (2006). Biology and biochemistry of glucosinolates. Annu. Rev. Plant Biol. 57, 303–333 10.1146/annurev.arplant.57.032905.105228 16669764

[B17] Hansen C. H. Wittstock U. Olsen C. E. Hick A. J. Pickett J. A. Halkier B. A. (2001). Cytochrome p450 CYP79F1 from Arabidopsis catalyzes the conversion of dihomomethionine and trihomomethionine to the corresponding aldoximes in the biosynthesis of aliphatic glucosinolates. J. Biol. Chem. 276, 11078–11085 10.1074/jbc.M010123200 11133994

[B18] Hanzawa Y. Imai A. Michael A. J. Komeda Y. Takahashi T. (2002). Characterization of the spermidine synthase-related gene family in *Arabidopsis thaliana*. FEBS Lett. 527, 176–180 10.1016/s0014-5793(02)03217-9 12220656

[B19] He Z. H. He D. Kohorn B. D. (1998). Requirement for the induced expression of a cell wall associated receptor kinase for survival during the pathogen response. Plant J. 14, 55–63 10.1046/j.1365-313X.1998.00092.x 9681026

[B20] Ishiguro S. Kawai-Oda A. Ueda J. Nishida I. Okada K. (2001). The DEFECTIVE IN ANTHER DEHISCIENCE gene encodes a novel phospholipase A1 catalyzing the initial step of jasmonic acid biosynthesis, which synchronizes pollen maturation, anther dehiscence, and flower opening in Arabidopsis. Plant Cell 13, 2191–2209 10.1105/tpc.010192 11595796 PMC139153

[B21] Janowitz T. Kneifel H. Piotrowski M. (2003). Identification and characterization of plant agmatine iminohydrolase, the last missing link in polyamine biosynthesis of plants. FEBS Lett. 544, 258–261 10.1016/s0014-5793(03)00515-5 12782327

[B22] Kakehi J.-I. Kuwashiro Y. Niitsu M. Takahashi T. (2008). Thermospermine is required for stem elongation in *Arabidopsis thaliana*. Plant Cell Physiol. 49, 1342–1349 10.1093/pcp/pcn109 18669523

[B23] King E. O. Ward M. K. Raney D. E. (1954). Two simple media for the demonstration of pyocyanin and fluorescin. J. Lab. Clin. Med. 44, 301–307 13184240

[B24] Knott J. M. Römer P. Sumper M. (2007). Putative spermine synthases from *Thalassiosira pseudonana* and *Arabidopsis thaliana* synthesize thermospermine rather than spermine. FEBS Lett. 581, 3081–3086 10.1016/j.febslet.2007.05.074 17560575

[B25] Kubigsteltig I. Laudert D. Weiler E. W. (1999). Structure and regulation of the *Arabidopsis thaliana* allene oxide synthase gene. Planta 208, 463–471 10.1007/s004250050583 10420644

[B26] Kusano T. Berberich T. Tateda C. Takahashi Y. (2008). Polyamines: essential factors for growth and survival. Planta 228, 367–381 10.1007/s00425-008-0772-7 18594857

[B27] Livak K. J. Schmittgen T. D. (2001). Analysis of relative gene expression data using real-time quantitative PCR and the 2-[Delta][Delta]CT method. Methods 25, 402–408 10.1006/meth.2001.1262 11846609

[B28] Maleck K. Levine A. Eulgem T. Morgan A. Schmid J. Lawton K. A. (2000). The transcriptome of *Arabidopsis thaliana* during systemic acquired resistance. Nat. Genet. 26, 403–410 10.1038/82521 11101835

[B29] Marco F. Alcazar R. Tiburcio A. F. Carrasco P. (2011). Interactions between polyamines and abiotic stress pathway responses unraveled by transcriptome analysis of polyamine overproducers. OMICS 15, 775–781 10.1089/omi.2011.0084 22011340 PMC3229227

[B30] Marina M. Maiale S. J. Rossi F. R. Romero M. F. Rivas E. I. Gárriz A. (2008). Apoplastic polyamine oxidation plays different roles in local responses of tobacco to infection by the necrotrophic fungus *Sclerotinia sclerotiorum* and the biotrophic bacterium *Pseudomonas viridiflava*. Plant Physiol. 147, 2164–2178 10.1104/pp.108.122614 18583531 PMC2492638

[B31] Marini F. Betti L. Scaramagli S. Biondi S. Torrigiani P. (2001). Polyamine metabolism is upregulated in response to tobacco mosaic virus in hypersensitive, but not in susceptible, tobacco. New Phytol. 149, 301–309 10.1046/j.1469-8137.2001.00017.x 33874627

[B32] Melotto M. Underwood W. Koczan J. Nomura K. He S. Y. (2006). Plant stomata function in innate immunity against bacterial invasion. Cell 126, 969–980 10.1016/j.cell.2006.06.054 16959575

[B33] Mikkelsen M. D. Petersen B. L. Glawischnig E. Jensen A. B. Andreasson E. Halkier B. A. (2003). Modulation of CYP79 genes and glucosinolate profiles in Arabidopsis by defense signaling pathways. Plant Physiol. 131, 298–308 10.1104/pp.011015 12529537 PMC166809

[B34] Mitsuya Y. Takahashi Y. Berberich T. Miyazaki A. Matsumura H. Takahashi H. (2009). Spermine signaling plays a significant role in the defense response of *Arabidopsis thaliana* to cucumber mosaic virus. J. Plant Physiol. 166, 626–643 10.1016/j.jplph.2008.08.006 18922600

[B35] Mitsuya Y. Takahashi Y. Uehara Y. Berberich T. Miyazaki A. Takahashi H. (2007). Identification of a novel Cys2/His2-type zinc-finger protein as a component of a spermine-signaling pathway in tobacco. J. Plant Physiol. 164, 785–793 10.1016/j.jplph.2008.08.006 16882456

[B36] Moschou P. N. Sarris P. F. Skandalis N. Andriopoulou A. H. Paschalidis K. A. Panopoulos N. J. (2009). Engineered polyamine catabolism preinduces tolerance of tobacco to bacteria and oomycetes. Plant Physiol. 149, 1970–1981 10.1104/pp.108.134932 19218362 PMC2663742

[B37] Moschou P. N. Wu J. Cona A. Tavladoraki P. Angelini R. Roubelakis-Angelakis K. A. (2012). The polyamines and their catabolic products are significant players in the turnover of nitrogenous molecules in plants. J. Exp. Bot. 63, 5003–5015 10.1093/jxb/ers202 22936828

[B38] Murashige T. Skoog F. (1962). A revised medium for rapid growth and bio assays with tobacco tissue cultures. Physiol. Plant. 15, 473–497 10.1111/j.1399-3054.1962.tb08052.x

[B39] Ozawa R. Bertea C. M. Foti M. Narayana R. Arimura G. Muroi A. (2009). Exogenous polyamines elicit herbivore-induced volatiles in lima bean leaves: involvement of calcium, H2O2 and Jasmonic acid. Plant Cell Physiol. 50, 2183–2199 10.1093/pcp/pcp153 19884250

[B40] Panicot M. Minguet E. G. Ferrando A. Alcazar R. Blazquez M. A. Carbonell J. (2002). A polyamine metabolon involving aminopropyl transferase complexes in Arabidopsis. Plant Cell 14, 2539–2551 10.1105/tpc.004077 12368503 PMC151234

[B41] Piotrowski M. Janowitz T. Kneifel H. (2003). Plant C-N hydrolases and the identification of a plant N-carbamoylputrescine amidohydrolase involved in polyamine biosynthesis. J. Biol. Chem. 278, 1708–1712 10.1074/jbc.M205699200 12435743

[B42] Planas-Portell J. Gallart M. Tiburcio A. F. Altabella T. (2013). Copper-containing amine oxidases contribute to terminal polyamine oxidation in peroxisomes and apoplast of *Arabidopsis thaliana*. BMC Plant Biol. 13:109 10.1186/1471-2229-13-109 23915037 PMC3751259

[B43] Schenk P. M. Kazan K. Wilson I. Anderson J. P. Richmond T. Somerville S. C. (2000). Coordinated plant defense responses in Arabidopsis revealed by microarray analysis. Proc. Natl. Acad. Sci. U.S.A. 97, 11655–11660 10.1073/pnas.97.21.11655 11027363 PMC17256

[B44] Seo H. S. Song J. T. Cheong J. J. Lee Y. H. Lee Y. W. Hwang I. (2001). Jasmonic acid carboxyl methyltransferase: a key enzyme for jasmonate-regulated plant responses. Proc. Natl. Acad. Sci. U.S.A. 98, 4788–4793 10.1073/pnas.081557298 11287667 PMC31912

[B45] Stenzel I. Hause B. Miersch O. Kurz T. Maucher H. Weichert H. (2003). Jasmonate biosynthesis and the allene oxide cyclase family of *Arabidopsis thaliana*. Plant Mol. Biol. 51, 895–911 10.1023/A:1023049319723 12777050

[B46] Takahashi T. Kakehi J.-I. (2010). Polyamines: ubiquitous polycations with unique roles in growth and stress responses. Ann. Bot. 105, 1–6 10.1093/aob/mcp259 19828463 PMC2794062

[B47] Takahashi Y. Berberich T. Miyazaki A. Seo S. Ohashi Y. Kusano T. (2003). Spermine signalling in tobacco: activation of mitogen-activated protein kinases by spermine is mediated through mitochondrial dysfunction. Plant J. 36, 820–829 10.1046/j.1365-313X.2003.01923.x 14675447

[B48] Takahashi Y. Berberich T. Yamashita K. Uehara Y. Miyazaki A. Kusano T. (2004). Identification of tobacco HIN1 and two closely related genes as spermine-responsive genes and their differential expression during the tobacco mosaic virus-induced hypersensitive response and during leaf- and flower-senescence. Plant Mol. Biol. 54, 613–622 10.1023/B:PLAN.0000038276.95539.39 15316293

[B49] Takahashi Y. Cong R. Sagor G. H. Niitsu M. Berberich T. Kusano T. (2010). Characterization of five polyamine oxidase isoforms in *Arabidopsis thaliana*. Plant Cell Rep. 29, 955–965 10.1007/s00299-010-0881-1 20532512

[B50] Tör M. Gordon P. Cuzick A. Eulgem T. Sinapidou E. Mert-Türk F. (2002). Arabidopsis SGT1b is required for defense signaling conferred by several downy mildew resistance genes. Plant Cell 14, 993–1003 10.1105/tpc.001123 12034892 PMC150602

[B51] Uknes S. Mauch-Mani B. Moyer M. Potter S. Williams S. Dincher S. (1992). Acquired resistance in Arabidopsis. Plant Cell 4, 645–656 10.1105/tpc.4.6.645 1392589 PMC160161

[B52] Urano K. Yoshiba Y. Nanjo T. Igarashi Y. Seki M. Sekiguchi F. (2003). Characterization of Arabidopsis genes involved in biosynthesis of polyamines in abiotic stress responses and developmental stages. Plant Cell Environ. 26, 1917–1926 10.1046/j.1365-3040.2003.01108.x

[B53] Van Loon L. C. Rep M. Pieterse C. M. (2006). Significance of inducible defense-related proteins in infected plants. Annu. Rev. Phytopathol. 44, 135–162 10.1146/annurev.phyto.44.070505.143425 16602946

[B54] Verica J. A. He Z. H. (2002). The cell wall-associated kinase (WAK) and WAK-like kinase gene family. Plant Physiol. 129, 455–459 10.1104/pp.011028 12068092 PMC1540232

[B55] Walters D. (2003a). Resistance to plant pathogens: possible roles for free polyamines and polyamine catabolism. New Phytol. 159, 109–115 10.1046/j.1469-8137.2003.00802.x 33873679

[B56] Walters D. Cowley T. Mitchell A. (2002). Methyl jasmonate alters polyamine metabolism and induces systemic protection against powdery mildew infection in barley seedlings. J. Exp. Bot. 53, 747–756 10.1093/jexbot/53.369.747 11886895

[B57] Walters D. R. (2003b). Polyamines and plant disease. Phytochemistry 64, 97–107 10.1016/S0031-9422(03)00329-7 12946408

[B58] Watson M. B. Malmberg R. L. (1996). Regulation of *Arabidopsis thaliana* (L.) Heynh arginine decarboxylase by potassium deficiency stress. Plant Physiol. 111, 1077–1083 10.1104/pp.111.4.1077 8756495 PMC160983

[B59] Watson M. W. Yu W. Galloway G. L. Malmberg R. L. (1997). Isolation and characterization of a second arginine decarboxylase cDNA from Arabidopsis (Accession No AF009647). Plant Physiol. 114, 1569 10.1104/pp.114.4.1567

[B60] Yamakawa H. Kamada H. Satoh M. Ohashi Y. (1998). Spermine is a salicylate-independent endogenous inducer for both tobacco acidic pathogenesis-related proteins and resistance against tobacco mosaic virus infection. Plant Physiol. 118, 1213–1222 10.1104/pp.118.4.1213 9847095 PMC34737

[B61] Zeier J. Delledonne M. Mishina T. Severi E. Sonoda M. Lamb C. (2004). Genetic elucidation of nitric oxide signaling in incompatible plant-pathogen interactions. Plant Physiol. 136, 2875–2886 10.1104/pp.104.042499 15347797 PMC523349

[B62] Zipfel C. Robatzek S. Navarro L. Oakeley E. J. Jones J. D. Felix G. (2004). Bacterial disease resistance in Arabidopsis through flagellin perception. Nature 428, 764–767 10.1038/nature02485 15085136

